# Comprehensive transcriptome analysis of the highly complex *Pisum sativum *genome using next generation sequencing

**DOI:** 10.1186/1471-2164-12-227

**Published:** 2011-05-11

**Authors:** Susanne U Franssen, Roshan P Shrestha, Andrea Bräutigam, Erich Bornberg-Bauer, Andreas PM Weber

**Affiliations:** 1 Institute for Evolution and Biodiversity, Westfalian Wilhelms University, Hüfferstrasse 1, 48149 Münster, Germany; 2 Department of Plant Biology, Michigan State University, 48823 East Lansing, MI, USA; 3 Institute of Plant Biochemistry, Heinrich Heine University, Universitätsstrasse 1, 40225 Düsseldorf, Germany

## Abstract

**Background:**

The garden pea, *Pisum sativum*, is among the best-investigated legume plants and of significant agro-commercial relevance. *Pisum sativum *has a large and complex genome and accordingly few comprehensive genomic resources exist.

**Results:**

We analyzed the pea transcriptome at the highest possible amount of accuracy by current technology. We used next generation sequencing with the Roche/454 platform and evaluated and compared a variety of approaches, including diverse tissue libraries, normalization, alternative sequencing technologies, saturation estimation and diverse assembly strategies. We generated libraries from flowers, leaves, cotyledons, epi- and hypocotyl, and etiolated and light treated etiolated seedlings, comprising a total of 450 megabases. Libraries were assembled into 324,428 unigenes in a first pass assembly.

A second pass assembly reduced the amount to 81,449 unigenes but caused a significant number of chimeras. Analyses of the assemblies identified the assembly step as a major possibility for improvement. By recording frequencies of Arabidopsis orthologs hit by randomly drawn reads and fitting parameters of the saturation curve we concluded that sequencing was exhaustive. For leaf libraries we found normalization allows partial recovery of expression strength aside the desired effect of increased coverage. Based on theoretical and biological considerations we concluded that the sequence reads in the database tagged the vast majority of transcripts in the aerial tissues. A pathway representation analysis showed the merits of sampling multiple aerial tissues to increase the number of tagged genes. All results have been made available as a fully annotated database in fasta format.

**Conclusions:**

We conclude that the approach taken resulted in a high quality - dataset which serves well as a first comprehensive reference set for the model legume pea. We suggest future deep sequencing transcriptome projects of species lacking a genomics backbone will need to concentrate mainly on resolving the issues of redundancy and paralogy during transcriptome assembly.

## Background

*Pisum sativum *(var. Little Marvel) is a legume of agro-commercial relevance [1] with a large genome, 4300 Mb [2], which is approximately five to ten times larger than that of *Medicago *[3]. At least a third but possibly more than half of the genome may consist of repetitive elements [4]. The garden pea was established as a biochemical model since it is easy to cultivate and fast growing. Additionally, unlike the *Brassicaceae *[5], it is low in glucosinolates, which interfere with enzyme activity and organelle intactness during isolation. Consequently, a large body of work on enzymes and organelles was carried out using pea as the model system, e.g. [5-11]. The presence of a sequenced genome or transcriptome is a massive advantage for the analysis of a model system with *Arabidopsis thaliana *providing the best example as the first plant model with genomic resources [12]. In the absence of a completely sequenced genome, plant EST collections, such as unigenes at NCBI [13], or tentative consensus sequences at DFCI [14], produced by traditional Sanger sequencing have proven extremely useful for plant research, e.g. [15].

Recently, it has become feasible to produce transcriptomic resources for non-model species by next generation sequencing (NGS) at reasonable cost. Next generation sequencing was employed to create transcriptome databases of species without a sequenced genome such as mangroves [16], eucalyptus [17], olive [18], chestnut [19] and *Artemisia annua *[20]. In all these projects 454/Roche NGS technology (reviewed in [21]) was used. For this RNAseq approach either fragmented mRNA or fragmented cDNA [22] can be used as input and read lengths ranging from 100 nucleotides (nts), 250 nts and 500 nts modal length can be received depending on the sequencer and sequencing kit employed, GS 20, GS FLX Standard Series and GS FLX Titanium Series, respectively (reviewed in [21,23]). In the various projects different assemblers were used, employing both overlap based methods [18,20] and strategies using de Bruijn graphs [16,17,19,24], either alone or in combination. Independent of the assembler used, the contigs obtained remained fairly short compared to traditional assemblies performed with Sanger reads: For GS20 data the average contig length was 130 bases for Eucalyptus [17] and 168 bases for chestnut [19]; for GS FLX reads of up to 250 bases the average contig length was between 334 bases and 433 bases [16-20]. Currently, new tools are developed for de novo assembly of these EST sequences since they are considerably shorter than ESTs generated by traditional Sanger sequencing, for example a version 3.0 of the overlap based assembler MIRA [25], the GS De Novo Assembler (alias Newbler) developed and provided by Roche/454 Life Sciences [23], both designed for the longer NGS reads (Roche/454) or Velvet designed for shorter NGS reads (e.g. Illumina) based on de Bruijn graphs [26]. To our knowledge, there is currently no standard as to how 454 reads are best assembled. After assembly, the resulting EST contigs and singletons ('unigenes') were annotated using publicly available databases and analyzed further for their biological information [16-20].

We chose to develop a transcriptome resource for *Pisum sativum *to (i) facilitate future biochemical, physiological, and cell biological experiments in *P. sativum *and (ii) evaluate different methods for generating sequence resources for non-model species with large and complex genomes. The different sequencing and assembly strategies were explored with respect to their potential for gene discovery and assessment of completeness. A transcriptome resource of the pea will greatly facilitate molecular and -omics approaches for research on this legume.

## Results and Discussion

### The sequence read databases yielded 450 Megabases of sequence

*De novo *sequencing of transcriptomes depends on sequencing technology with long read lengths such as 454/Roche technologies to facilitate EST assembly without a genomic reference [27]. To generate a pea transcriptome database, four cDNA libraries of cotyledons, etiolated seedlings, etiolated seedlings exposed to light for 6 hours, and epicotyl were normalized and sequenced with GS FLX technology yielding average raw read lengths of 236 nts and an average trimmed read length of 228 nucleotides (nts) (Table 1). GS FLX sequencing yielded more than a quarter billion nts after removing primers from the raw reads (Table 1). Six cDNA libraries of flowers, hypocotyl and four leaf cDNA libraries were normalized and sequenced with GS20 technology which resulted in an average raw read length of 103 nts and an average trimmed read length of 97 nts with more than 150 million nts sequenced (Table 1). The read length for both sequencing technologies were in agreement with predicted read lengths [23]. The raw reads were trimmed and cleaned with cross match [28] and MIRA [25] to a total of 2,209,735 final reads for assembly (Table 1). Singleton reads that had no apparent overlap with other reads in the database were sorted out and deposited into a "debris list" by MIRA [25,29]. These singletons were blasted against TAIR9 and 189,510 out of the 806,194 debris reads which matched to an *Arabidopsis *gene (BlastX, e-value ≤ 10^-4^) were included in the following analysis. Based on the contig annotation percentage, we estimated that approximately 40,000 additional reads were true singletons identifying different transcripts, but there is currently no method to retrieve them from the MIRA single read bin. If the pea genome was sequenced, these additional reads could be retrieved from the read database by mapping them to the genome, as has been done for maize 454 reads [30].

**Table 1:** Properties of the different libraries; abbreviations are as follows: COT cotyledons, E etiolated leaves, L light treated etiolated leaves, EPI epicotyl, LVN.1-5 leaf libraries 1-5, FLO flower, HYP hypocotyl, LVR.1 leaf library, non normalized; ^a^sequenced with GS flex, ^b^sequenced with GS 20; all libraries were normalized except LVR.1;

library	number of raw reads	number of reads after crossmatch clean up	number of reads after MIRA	number reads for the assembly	number of reads with AGI mapping	raw nts	nts after crossmatch	mean readlength raw	mean read length after crossmatch
COT^a^	343,694	335,050	272,731	272402	220,875	81,050,491	75,679,454	235	225
E^a^	144,290	135,906	120,202	120126	101,119	34,849,428	32,229,999	241	237
L^a^	192,117	174,563	157,977	157921	137,359	46,654,732	41,408,162	242	237
EPI^a^	243,294	233,143	189,093	188193	148,059	54,072,084	50,193,810	222	215
LVN.5^a^	320,209	311,538	268,315	267966	223,735	77,473,643	72,627,353	241	233

sum/average^a^						294,100,378	272,138,778	236	228
									
LVN.1^b^	350,016	343,361	278,080	277888	228,894	36,551,286	33,767,205	104	98
LVN.2^b^	302,341	295,416	234,029	233790	192,903	31,399,731	29,009,144	103	98
LVN.3^b^	146,504	142,862	115,832	115751	96,087	15,049,571	13,963,252	102	97
LVN.4^b^	278,936	272,367	223,939	223596	184,175	28,590,490	26,562,093	102	97
FLO^b^	162,353	153,765	95,229	95097	70,957	17,047,889	15,039,288	105	97
HYP^b^	349,976	327,769	255,437	255310	210,073	36,269,788	31,823,328	103	97

sum/average^b^						164,908,755	150,164,310	103	97
									
LVR.1^b^	308,042	293,201	191,394	191205	167,551	27,779,787	24,062,060	90	82

total	3,141,772	3,018,941	2,402,258	2399245		486,788,920	446,365,148		

### Two assemblies had very different properties

**Figure 1:**
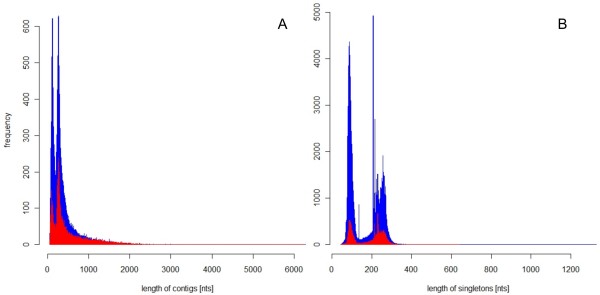
**Length distribution of contigs (A) and singletons (B)**. Length distribution of unigenes obtained from the first pass MIRA assembly (blue) and the additional second pass assembly with TGICL including CAP3 (red). (A) Length distribution of all contigs. There are 128,767 contigs with the largest contig size of 6283 nucleotides in the MIRA assembly and 45,686 contigs after the second pass TGIGL/CAP3 assembly with the largest contig size of 6258 nucleotides. (B) Length distribution of all singletons that were employed for subsequent analysis. The MIRA assembly resulted in 195,661 singletons, 35,763 singletons remained after TGICL/CAP3 assembly.

The cleaned and trimmed reads were assembled in a two pass assembly. The initial assembly was done with MIRA followed by a second pass assembly with the TGICL pipeline that includes the CAP3 assembler. The results of the second pass assembly are available as additional file 1. The final reads were assembled with MIRA to 128,767 contigs with the largest contig covering 6283 bp. The contig length distribution showed a bimodal pattern with two peaks at 108 nts and 260 nts that were a result of the two different sequencing platforms employed (Figure 1A). The mean contig length was 324 bases, which was comparable to the mean contig lengths of other NGS projects of plant transcriptomes reflecting comparable assembly performance [16-20]. Besides the low confidence debris reads MIRA also created singleton reads of higher confidence. These higher confidence singletons together with the previously described 189,510 reads recovered from the debris added up to a total of 195,661 singletons, which were considered in the subsequent analysis. The singleton length distribution had two peaks shifted to lower numbers of 88 and 208 nts with the two peaks representing the two sequencing platforms employed (Figure 1B).

While MIRA was a conservative assembly program [29] CAP3 and phrap have been used to assemble a transcriptome [16,24] and can create a more lenient assembly. A second less stringent assembly was produced from the MIRA contigs using the TGICL pipeline which includes CAP3 [31,32]. Relaxed parameters of 40 bases overlap and ≥94% identity were chosen because these parameters were used for assembling mangrove transcriptomes [16]. This resulted in a total of 81,449 unigenes, reducing the number of singletons to 18% and the number of contigs to 35% of the original number of singletons and contigs, respectively. The mean contig length increased to 454 bases. Although the number of singletons and short contigs decreased in the second pass assembly, the number of large contigs did not increase (Figure 1A). To analyze whether the assemblies only reduced redundancy or whether they also created chimeric contigs, the initial reads, the contigs of the first pass assembly, and the contigs of the second pass assembly were mapped against the transcriptomes of the model species *Arabidopsis thaliana*, *Glycine max *(soy bean) and *Medicago truncatula*. The reads themselves mapped to 18,856 genes of *A. thaliana*; the unigenes of the first pass assembly mapped to 90.4% while the unigenes of second pass assembly only mapped to 62.2% of the originally found genes. Mappings to *G. max *and to *M. truncatula *yielded similar results in that the second pass assembly lost about a third of contig annotations obtained for the first pass assembly (Additional File 2). The reduced identification of genes by the second pass unigenes for the various references indicated that the first pass assembly massively decreased redundancy while leading only to a modest decrease in matchable reference genes. The lenient second pass assembly lead to a further reduction in redundancy (Figure 1A) but also created chimeric unigenes joining sequences originating from different transcripts indicated by the loss of one third of the identified genes. It was also attempted to benchmark a MIRA assembly performed with 454 reads only against Sanger sequencing generated coding sequences from public sources. However, there were only 2,281 partial and complete coding sequences of the garden pea available at NCBI [13]. Comparison of these sequences to the contigs yielded no conclusions beyond the comparison to more distant reference genomes. It was also attempted to use de Bruijn graph based assemblers such as velvet [26] and SOAP [33] for 454 read based transcriptome assembly, however the contig length distribution was inferior to the overlap based assemblers (data not shown) and the analysis was not pursued further.

All subsequent analyses were thus based on the MIRA first pass assembly unigenes. As the mappings to the three reference genomes yielded qualitatively similar results, we chose to base subsequent analyses on the *A. thaliana *reference as it is the plant genome which is currently best annotated.

### The database annotation revealed low contamination and high redundancy

**Figure 2:**
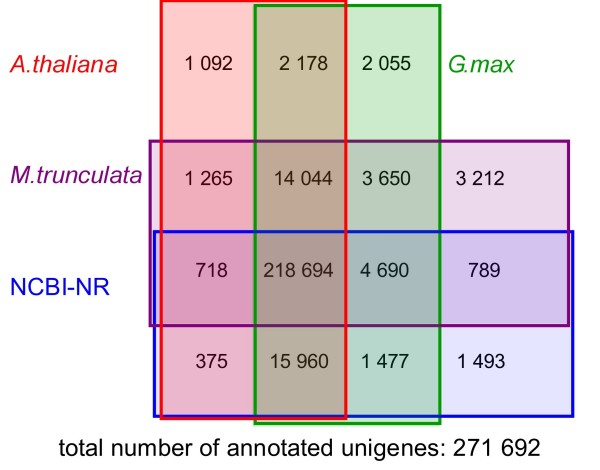
**Comparison of the number of MIRA unigene annotations obtained from the different reference databases (*A. thaliana*, *G. max*, *M. trunculata *and NCBI NR)**; For the annotation the best BlastX hit against the protein sequences of the reference organisms was employed with an e-value cut off of X ≤ 10^-4^. The total number of annotated unigenes is 271,692 corresponding to 84% of all unigenes.

To predict and analyze the function of the 324,428 unigenes of the first pass MIRA assembly, they were annotated with *A. thaliana *followed by *M. truncatula*, *G. max*, and the nr database of NCBI. 90% of these unigenes could be mapped to a sequence from either *A. thaliana*, *M. truncatula*, *G. max*, or NCBI's NR database with BlastX with an e-value ≤10^-4 ^(Figure 2). Only 0.5% of the reads that could be mapped to any of those protein resources could be exclusively mapped to NR (Figure 2). The contamination by plant pathogens of viral or bacterial origin or other non-plant sequences introduced during preparation was thus very low. Additionally the unigenes of both assemblies contained very limited sequences derived from repetitive elements in the genome (Additional File 3).

During the mapping, a group of AGIs were identified by the presence of multiple unigene matches from the unigene database (Table 2). 2,741 AGIs were hit more than 10 times with a maximal number of 27,525 hits on a gene encoding a light harvesting complex protein (Table 2) and 90 AGIs were hit more than 100 times. Of the ten most frequently hit AGIs, five were light harvesting complex encoding genes in addition to the small chain of RubisCO, a GTP binding protein, a ribosomal protein, and a protein of unknown function (Table 2). Based on the MIRA documentation [33,34], we interpreted this finding as a high degree of redundancy between some unigenes, which may be due to sequencing errors, paralogy, or allelism within a particular gene precluding assembly of unigenes into larger contigs. To test this assumption, we randomly chose several unigenes and used them to query all the redundant sequences with BlastN. This showed that some unigene sequence information is highly similar with sequence identities between 94-100% and identical changes were recurring in many sequences (data not shown). This phenomenon was also observed during the sequencing of the mangrove transcriptome conducted with more lenient assembly parameters [16]. In principle, there were several possible factors contributing to this observation: (i) sequencing errors resulting in difficulties during the assembly [25,29]; (ii) different alleles of the genes, which result in base changes and small insertions or deletions [25,29]; (iii) different splice variants that enforce assembly into different unigenes; (iv) assembly problems; (v) massive expansions of gene families in the garden pea [34,35], or a combination thereof.

**Table 2:** The ten most frequent AGIs that were used for the annotation of unigenes with BlastX (e-value ≤ 10^-4^)

# of AGI occurrences	AGI	Annotation
2658	AT5G18380	40S ribosomal protein S16 (RPS16C)
2684	AT5G54270	LHCB3 (LIGHT-HARVESTING CHLOROPHYLL B-BINDING PROTEIN 3)
3138	AT1G04280	unknown protein
3566	AT2G34430	LHB1B1; chlorophyll binding
4301	AT2G05070	LHCB2.2; chlorophyll binding
6464	AT5G20010	RAN-1; GTP binding/GTPase/protein binding
8636	AT1G67090	RBCS1A (RIBULOSE BISPHOSPHATE CARBOXYLASE SMALL CHAIN 1A)
15392	AT1G29930	CAB1 (CHLOROPHYLL A/B BINDING PROTEIN 1); chlorophyll binding
18815	AT3G27690	LHCB2.3; chlorophyll binding
27525	AT2G34420	LHB1B2; chlorophyll binding

To better distinguish between sequence variation of biological origin (i.e., alleles, paralogs) and of technical origin (i.e., sequencing and assembly errors), we retrieved Sanger sequenced cDNA for the five genes listed in Table 2 for which the sequence information was available at NCBI [17]. We tested how many positions of the cDNA were (i) identical among all matching unigenes, (ii) covered by at least 25 identical variants (identical point mutations) within the unigenes, (iii) covered by at least 4 insertions/deletions of a length dividable by three, and (iv) being covered by at least one variant (point mutation or insertions/deletions of a length not dividable by three) but not exceeding the required threshold of 25 identical variants (Table 3). Cases (ii) and (iii) we interpreted as putative positions containing biological variants that could differentiate alleles or paralogs, while case (iv) can be interpreted as putative positions containing sequencing errors. To strengthen the analysis, we added five additional genes, which had been published previously as single copy genes in the pea genome [36-40]. We further tested four single copy Mendelian genes, seed shape, seed color, flower colour, and size [41-44]. The BWA mapping gave no indication of longer insertions or deletions. The resulting alignments are summarized in Table 3 and can be viewed by loading additional files 4 and 5 into an alignment viewer such as tablet [45]. In principle, both allelic variation as well as paralogous genes are possible sources of biological variants mapping to the identical reference gene. However, the sequencing libraries used in this study were made from pooled tissues harvested from varying numbers of different plants that were all grown from a commercial seed source and not from single seed decent. Thus, the design of our study does not permit to distinguish allelic variation from paralogous genes, also keeping in mind that they are not full-length transcripts. Between 0.9 and 16.4% of the positions of the genes tested were covered by frequent (i.e. >25 or insertions/deletions dividable by 3) variants, which we took to represent the maximum amount of either allelic variation or paralogous, recently duplicated genes (Table 3). For the 9 known single copy genes analyzed in our study, the frequent variants were between 0.1 and 1.1% of the positions (Table 3). For these single copy genes, paralogous genes could be excluded as the source of this variation; hence these figures represent the maximum amount of positional allelic diversity, which we thus consider to be smaller than 1%. Recently, an analysis in the Brassicaceae *Cleome gynandra *on a smaller scale also identified genetic variation within transcript sequences [46]. The vast majority of variable positions were covered by infrequent variants, which we interpreted to represent sequencing errors and not variation of biological origin (Table 3). 454 sequencing technologies are known to produce sequencing errors of about 1%, especially in homopolymer stretches [23]. This error rate probably contributed to redundancies between unigenes especially as MIRA provided conservative assemblies [25,29]. We thus concluded that the large number of unigenes obtained in the MIRA assembly is predominantly due to sequencing errors, which hampered the assembly. Finally, although many of the unigenes were highly similar, we also found those with 100% identical overlaps that thus should have been collapsed during the assembly. Further, the list of genes with the high number of matching unigenes (Table 2) was notably full of genes expected to be highly expressed in the tissues sampled for library construction. Intuitively, those genes with the highest expression should yield the best resolved and longest unigenes as the gene coverage was highest. Thus a high negative or no correlation was expected between the number of reads and number of unigenes matching an AGI. Counter intuitively, the Spearman correlation coefficient was +0.75 for the first pass assembly and +0.71 for the second pass assembly: When a large number of reads matching a reference gene was present, also a large number of unigenes matching this reference gene was present (Additional File 6). This may indicate that both MIRA and CAP3 were unsuccessful in assembling especially those transcripts with high library representation (Additional File 6). To our knowledge, other transcriptome sequencing projects using the same assemblers did not report on this phenomenon [16-20], although a large number of unigenes matching to one reference was observed at least once [16]. Resolving this possible mapping problem will require assembling a 454 read set for a model species since this will allow tests against the complete reference genome. The correlation analysis points to the assembly as at least one of the reasons for the large number of unigenes recovered. We thus conclude that assembly and sequencing errors, but not expansion gene families and biological sequence variation contribute to the high number of largely redundant sequence contigs obtained in our study.

**Table 3:** Quantification of different sources contributing to redundancy between unigenes; unigenes where mapped against 14 cDNA reference sequences of *P. sativum*: orthologs of the ten most frequent AGIs (Table 2), five known single copy genes from *P. sativum*, and four genes encoding Mendelian traits; the original alignments can be viewed by loading additional files 4 and 5 into an alignment viewer such as tablet [45].

gene name	corresponding pea cDNA	length in bases	# reads mapped	identical positions	percentage	putative SNP positions	percentage	putative sequencing error positions	percentage
LHCb1	gi|56809378	801	56517	9	1.1	131	16.4	779	97.3
LHCb2	gi|56809380	798	15370	28	3.5	9	1.1	768	96.2
CCBP	gi|141448063	922	10616	609	66.1	18	2.0	296	32.1
Ran1	gi|123192430	666	6488	363	54.5	8	1.2	300	45.0
RuBP	gi|169152	674	651	182	27.0	68	10.1	475	70.5
ApxI	gi|169042	3625	32	1367	37.7	8	0.2	99	2.7
ek-oxidase	gi|37954113	1797	1474	1749	97.3	20	1.1	25	1.4
Fed-1	gi|169086	1995	35	1360	68.2	1	0.1	87	4.4
HMG1	gi|436423	807	6	670	83.0	5	0.6	124	15.4
plastocyanin	gi|20845	1505	107	1050	69.8	2	0.1	453	30.1
G3bh	gi|2316017	4248	3	3577	84.2	0	--	1	0.0
bHLH	gi|308084332	11892	0	0	--	nd	nd	nd	nd
SBEII	gi|510546	2919	14	2186	74.9	20	0.7	18	0.6
SGR	gi|156713218	792	1	787	99.4	4	0.5	1	0.1

### While the transcripts were well covered, the transcriptome coverage was limited

**Figure 3:**
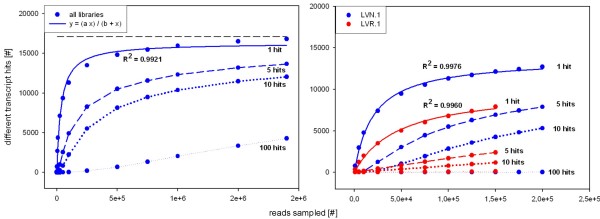
**Rarefaction analysis of gene representation in different libraries**; in each library different numbers of randomly sampled reads were blasted against Arabidopsis peptides (TAIR9) and the number of identified AGIs tagged at least once, five, ten and 100 times was recorded. The resulting data was modeled with non-linear regression fitting y = (ax)/(b+x) (continuous line). (A) Reads were randomly sampled from the union of all 12 libraries. The total number of different AGIs identified is 17 104. (B) Reads were randomly sampled from either normalized leaf library one (LVN.1, blue) or the non-normalized leaf library (LVR.1, red). The total numbers of different AGIs used for annotation for the normalized and the non-normalized library are 13,298 and 8,515, respectively. Data from all libraries is summarized in table 4 and plots are presented in the Additional file 7.

Two parameters define the quality of the database: the number of possible transcripts tagged by at least one read ('transcript coverage') and the number of possible bases covered ('transcriptome coverage'). The unigenes in the MIRA assembly corresponded to about 60% of the protein coding genes of *A. thaliana *[47]. This number was comparable to the number of genes detected in *A. thaliana *tissues by microarray experiments [48] indicating that the library preparation method captured the majority of transcripts in a tissue. For this comparison, however, it has to be kept in mind that both species are separated by about 90 million years of evolution since the split between Fabaceae and Brassicaceae [49], providing opportunities for mutation as well as small and large scale duplications changing the gene repertoire. We chose to also test the transcript coverage mathematically with a strategy similar to rarefaction analysis. A detailed description of the mathematical method is given in material and methods. Fitting the number of identified *Arabidopsis *genes for different samples sizes for the different libraries to hyperbolic curves resulted in the parameters summarized in table 4. The different libraries had slopes of 0.003 to 0.018 at the final read count (Table 4). Two leaf libraries, the cotyledon and the hypocotyl library had excellent coverage indicated by low slopes at the final read count, (Table 4, Figure 3, Additional file 7) which indicated that in these libraries almost all possible AGIs were detected. However, the flower library (FLO), the libraries from etiolated tissue (E and L) and one of the normalized leaf libraries (LVN.3) retained relatively high slopes at the final read count. This indicated that sequencing of a higher number of reads from those two libraries would have resulted in the identification of more genes in the respective libraries. The differences in the slope at the final read count between the different libraries was mostly due to a low final read count rather than differences in the fitted hyperbolic curves (Table 4).

**Table 4:** Properties of the different libraries; a and b, fitting parameters of the equation y = ax/(b+x) with 'a' representing the AGI (Arabidopsis genome identifier) detections maximally possible (for a detailed description please see material and methods)

library	a	b	R^2^	total AGIs detected	slope at final read count
COT	11629	35034	0.9948	10873	0.0043
E	13062	18865	0.9989	11566	0.0128
L	12734	20070	0.9987	11631	0.0081
EPI	13662	24062	0.998	12736	0.0073
LVN.5	13109	30514	0.9968	12283	0.0045
LVN.1	13715	20362	0.9976	13298	0.0031
LVN.2	13559	19746	0.9982	12981	0.0042
LVN.3	12718	16929	0.9995	11465	0.0122
LVN.4	13359	19496	0.9986	12670	0.0044
FLO	12382	18261	0.9998	10774	0.0176
HYP	13741	23566	0.997	13071	0.0042
LVR.1	10087	46963	0.996	8515	0.0084
all	16314	35169	0.9921	17104	0.0001

Since leaf libraries were analyzed with different sequencing technologies, GS20 and GS FLX, and with and without normalization, the effect of each factor on the transcript coverage could be assessed. In the normalized leaf libraries the detection of between 12,718 and 13,715 genes was the upper limit whereas in the non-normalized library only 10,087 AGIs could be identified based on the model and unlimited sequencing. Theoretically it should be possible to detect ESTs for lowly expressed genes even from a non-normalized library given unlimited sample sizes. Nevertheless the data clearly showed that with comparable library sizes the number of tagged genes was significantly increased in normalized libraries. The reason was probably that sequencing libraries were large but not unlimited. Hence very abundant leaf transcripts out-competed transcripts of low abundance for "sequencing space" in the library leading to the lower number of AGIs that could be identified in the non-normalized library. Possibly, cDNA synthesis primers got depleted by very abundant messages in non-normalized libraries, thereby leading to the suppression of less abundant transcripts in the sequencing library. GS20 sequenced libraries and GS FLX sequenced libraries yielded similar numbers of possible AGIs tagged by at least one read. Based on the mathematical analysis the majority of sequencing libraries were sequenced to exhaustion (Table 4).

It was more difficult to estimate the amount of bases covered in relation to all bases of the complete pea transcriptome (in other words the 'transcriptome coverage') since the genome of the garden pea has not been sequenced yet. To approximate the transcriptome coverage, the number of AGIs tagged by multiple reads was tested. Most transcripts will be longer than one read; thus coverage with multiple reads is required for complete bases coverage for most of the transcripts. Reads were again drawn from the read pool of a specific library/combination of all libraries with the number of AGIs identified by 5, 10 and 100 reads, respectively, recorded. In the combined library, a considerable slope remained at the final read number, indicating that respective fold coverage was reached only for a subpopulation of AGIs (Figure 3A). Since a subpopulation of AGIs was hit by only one read, the unigenes resulting from this subpopulation were expected to remain short; i.e., the coverage did not suffice for assembly despite the large number of sequenced reads (Table 1). In an alternative approach, BlastX combined with in house python scripts was used to determine the total sequence coverage for all tagged *Arabidopsis *proteins. The database of Medicago coding sequences [50] covered 35% of the Arabidopsis proteome; the database of *G. max *coding sequences [51] covered 49% of of the Arabidopsis proteome (based on BlastX, e-value ≤ 10^-4^). The pea unigene collection from this database covered 31% of the tagged AGIs. A simulation based on a fragmented Arabidopsis transcriptome estimates that with 450 Mb of bases, the complete transcriptome ought to be covered [52]. Since the percentage of the pea database mapping remained below both other legume mappings (31% to 35% and 49%, respectively), the approach based on BlastX supported the results of the tagging analysis: both analyses indicated that the transcriptome coverage was not complete. The gaps resulting from incomplete coverage probably precluded complete assembly and many short unigenes persisted (Figure 1). The missing coverage was not located at either the 5' or 3' end of the transcripts. The sequence read population was tested for 5' and 3' prime bias since the libraries were created with poly d(T) priming. The results indicated only little bias against the 5' end compared to the 3' end of the coding sequence (Additional file 8). Based on the transcript and transcriptome coverage, the number of sequence reads was sufficient to tag the majority of transcripts but not to assemble the transcriptome to completeness.

### Normalization has unexpected effects on the sequence recovery

The detailed analysis of the leaf libraries revealed that the normalization leads to increased coverage since a larger number of AGIs is identified by 5 and 10 reads, respectively (Figure 3B). Neither of the curves reached saturation, however, indicated by the considerable slope remaining at the final read count (Figure 3A, B). After establishing that even normalized libraries retained subpopulations of transcripts that were tagged by one read only and subpopulations that were tagged by up to 500 reads, we tested whether normalized libraries still retain quantitative information or whether normalization was complete. The Spearman rank coefficient was calculated pairwise for the non-normalized leaf libraries versus the normalized leaf library (Table 5). Despite the normalization, the Spearman rank coefficients varied between 0.697 for LVN.5, the only GS FLX sequenced leaf library, and 0.762 for LVN.1. This indicated a strong positive rank-correlation of expression between the normalized and non-normalized libraries. The correlation coefficients between the different normalized libraries ranged from 0.749 to 0.913 with the GS FLX sequenced library LVN.5 yielding the lowest coefficient (Table 5). The normalization was reproducible between libraries. Sequencing technology, however, was an important variable between libraries influencing the results as the only library sequenced with GS FLX technology yielded the weakest correlation for all (Table 5). It was attempted to recover expression profiles from normalized libraries by developing an algorithm that captured the transformation from a non-normalized to a normalized library using the six leaf libraries. This approach, however, was not successful.

**Table 5:** Correlation coefficients between expression profiles of the different normalized leaf libraries and the non-normalized leaf library; the expression was determined as the number of reads mapping to an AGI; R1 non normalized library LNR

compared libraries	Spearman's rank coefficient
R1 vs. N1	0.7620616
R1 vs. N2	0.7490598
R1 vs. N3	0.6996976
R1 vs. N4	0.7269965
R1 vs. N5	0.6974041

N1 vs. N2	0.9134052
N1 vs. N3	0.8691016
N1 vs. N4	0.8862503
N1 vs. N5	0.8048091
N2 vs. N3	0.8629818
N2 vs. N4	0.8800997
N2 vs. N5	0.7952054
N3 vs. N4	0.8360410
N3 vs. N5	0.7496826
N4 vs. N5	0.7811579

**Figure 4:**
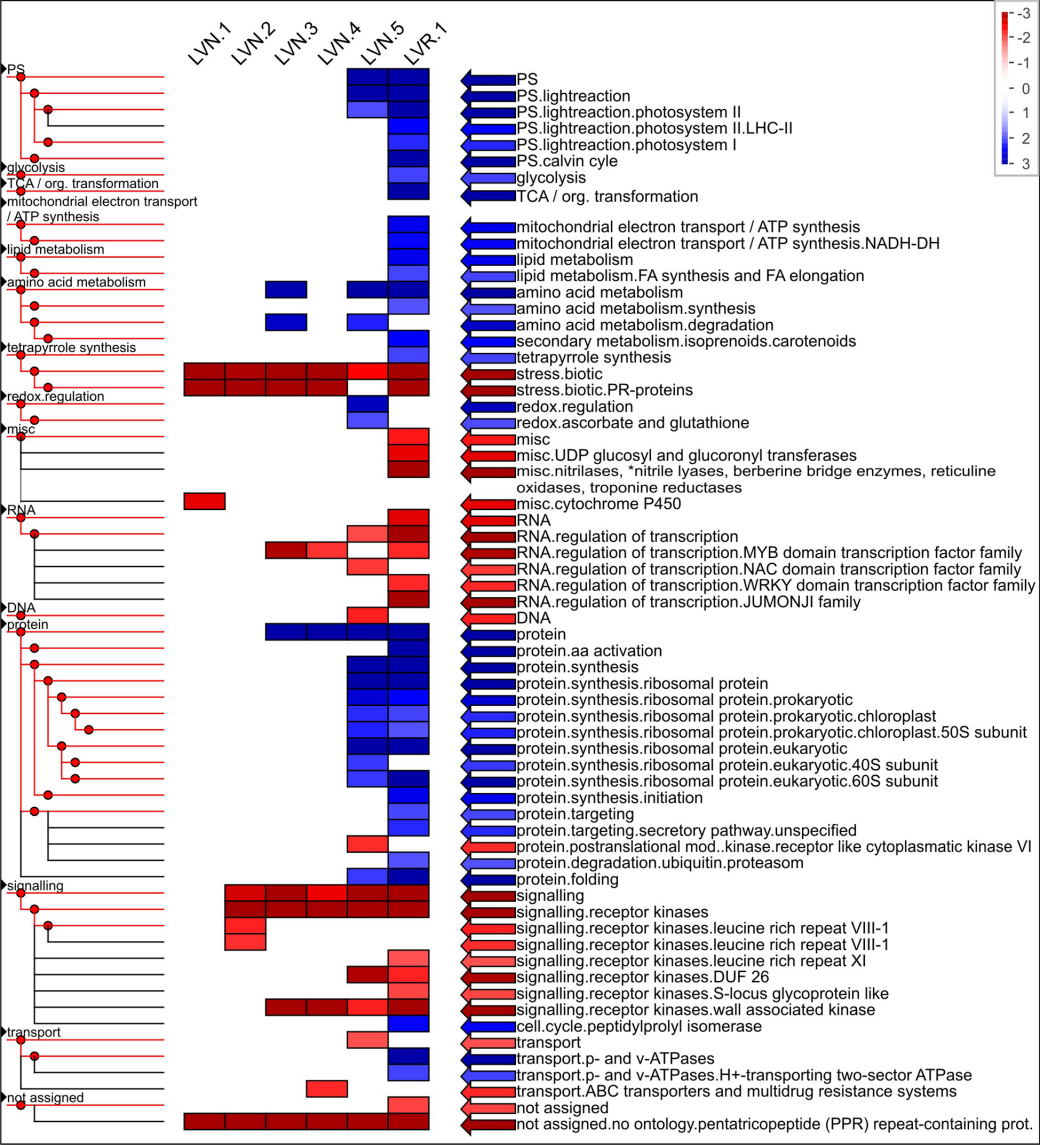
**Enriched terms of MapMan categories of normalized and non-normalized leaf libraries**; Enrichment of each category was tested with Fisher's exact test with counts for the categories of genes present in one library against the counts for the categories of genes present in the union of all leaf libraries from standard conditions. Red and blue boxes indicate categories that are overrepresented or underrepresented, respectively (α = 0.05). Multiple testing was corrected for by FDR (Benjamini-Hochberg). For the graphical representation the p-values for the different categories are transformed into z-scores, thus a p-value of 0.05 is assigned to a value of 1.96. The figure was created with PageMan software [59] and subsequently modified.

It was tested if normalization or lack thereof influenced the types of genes which were detected in the resulting libraries of the same tissue. All AGIs identified with at least one read were counted as present in the respective leaf libraries and enrichment of functional categories was analyzed with PageMan software (Figure 4) [53]. 63 MapMan categories [54] were enriched and/or depleted in any of the six leaf libraries. Thereof 51 categories were either over- or underrepresented in the non-normalized library. Major metabolic pathways such as photosynthesis, central carbon metabolism, energy metabolism as well as lipid, amino acid, and protein biosynthesis were overrepresented whereas stress, regulation of transcription, and signaling categories were underrepresented. The underrepresented categories contained genes which were mainly lowly expressed in plants [55] and thus were probably underrepresented if the library was not normalized. However, deeper sequencing, i.e. sequencing of higher read numbers, may not be a solution to recovering those genes in non-normalized libraries as the hyperbolic curves fitted to the data indicated that only 75% of genes present in normalized leaf libraries could be recovered from non-normalized libraries (Table 4). Thus, although massively parallel sequencing allowed better coverage and quantification of lowly expressed genes [56,57], it may still not cover all expressed genes in a library of defined volume unless normalization is applied. Among the normalized libraries, the library LVN.5 which was sequenced with GS FLX and not with GS 20 had a different pattern compared to the other normalized libraries with more categories enriched and depleted. In this library, also fewer Arabidopsis genes were tagged by reads (Table 4) although the absolute read count was comparable or higher than that of the other leaf libraries (Table 1). This may suggest less efficient normalization, but the Spearman rank coefficient for library LVN.5 vs. the non-normalized library LVR.1 was lower than those of the other normalized libraries compared to the non-normalized one (Table 5). We thus suspect that there was a biological difference between non-normalized leaf library five and the other normalized leaf libraries that changed the pathway representation patterns. Alternatively, sequencing with a different technology may have changed the pattern in an unforeseen way.

### The different libraries had different pathway representations

**Figure 5:**
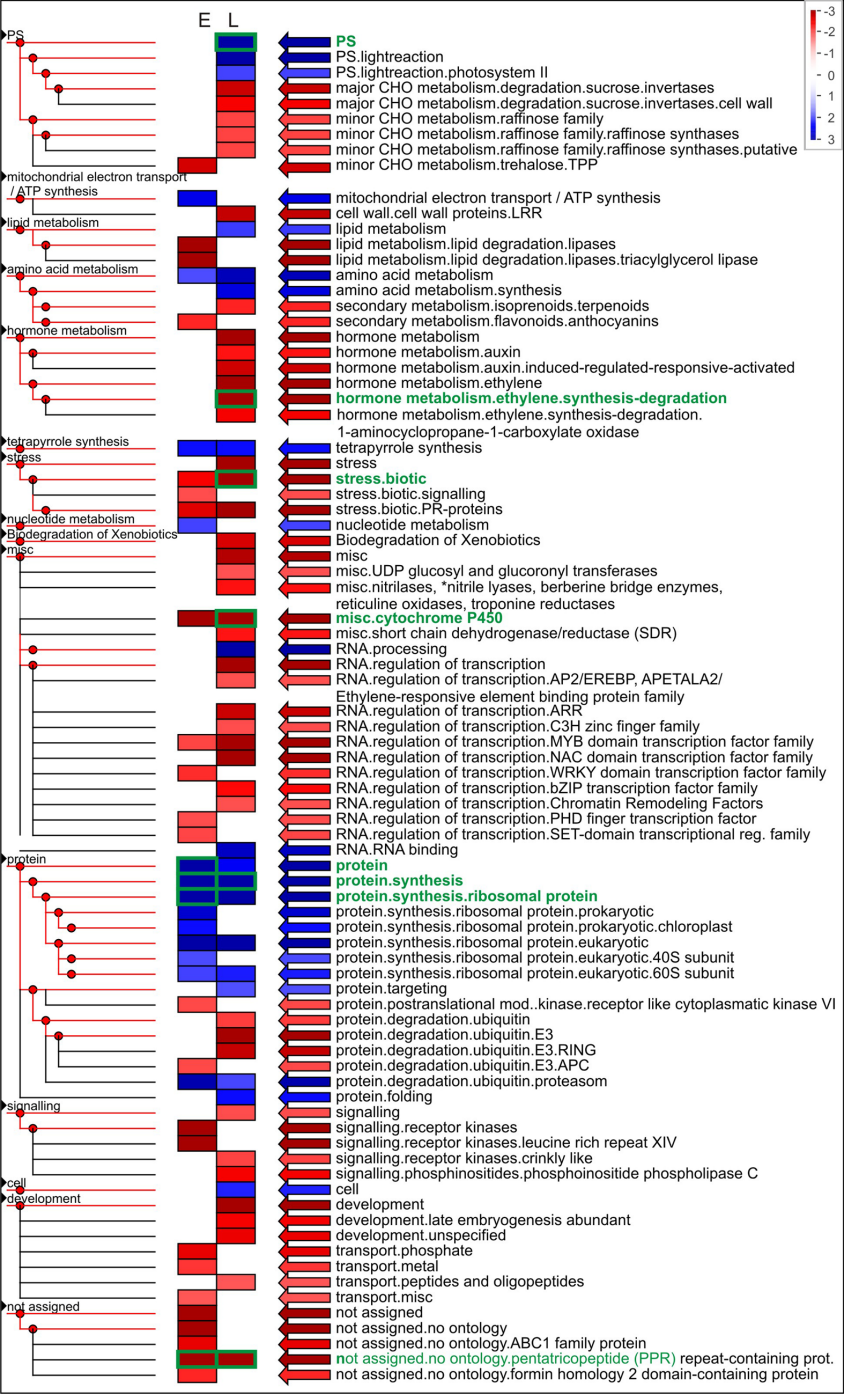
**Enriched terms of MapMan categories for libraries of etiolated seedlings and etiolated seedlings after light treatment**; enrichment was tested as described for Figure 4 except the figure shows results without multiple testing correction, the categories that passed multiple testing correction (by FDR, Benjamini-Hochberg) are marked in green.

Finally, we hypothesized that sequencing multiple aerial tissues under different conditions will lead to broader coverage of metabolic pathways and cellular processes. This hypothesis was tested with the pathway distribution in the different libraries. We compared two sequencing libraries which were isolated from etiolated seedlings grown under identical conditions except for a 6 h light treatment (i.e., de-etiolation) before harvesting. The metabolic pathways and cellular processes were represented by MapMan categories [54] and visualized with PageMan Software (Figure 5) [53]; GO term enrichment was tested with topGO [58,59]. MapMan categories were hierarchical [54] and thus ideally suited to our question. The light-treated library contained more over- and underrepresented pathways compared to the non-treated library. In light-treated samples, 59 of 82 categories were either enriched or depleted, whereas in non-light treated samples this was only the case for 37 out of the 82 categories. The library from etiolated seedlings was enriched in mitochondrial electron transfer, amino acid metabolism, tetrapyrrol and nucleotide biosynthesis, protein synthesis, and ubiquitin mediated protein degradation. This etiolated seedling pathway profile represented a heterotrophic tissue poised to differentiate upon an environmental cue. The overrepresentation of mitochondrial metabolism and amino acid metabolism reflected the dependence on stored metabolites, possibly amino acids, for energy [60]. Several anabolic pathways such as tetrapyrrol biosynthesis, nucleotide synthesis, and the protein category were also overrepresented. Once the seedlings are exposed to a short term light impulse, the pathway profile shifted. The light-treated library was enriched in the categories of photosynthesis, lipid and amino acid metabolism, tetrapyrrol biosynthesis, protein synthesis, ubiquitin mediated protein degradation, protein folding, and the category cell. After the light treatment, plants initiated a program to switch to photoautotrophic growth [61]. Photosynthetic genes, especially of the light reaction, were qualitatively overrepresented in the library. The remaining overrepresented categories reflected the buildup of the photosynthetic apparatus in the thylakoid membranes for photosynthesis and the shift away from a heterotrophic lifestyle upon light exposure [61]. The mitochondrial electron transfer category was no longer overrepresented. In addition to the anabolic pathways already overrepresented in etiolated seedlings, the light treated library was overrepresented in the categories of lipid metabolism, amino acid synthesis, protein targeting and protein folding reflecting the needs for thylakoid synthesis and assembly. The massive induction of photosynthesis genes had also been reported for the photomorphogenic transition of Arabidopsis [61]. We also tested the corresponding non-hierarchical GO terms for enrichment but found less striking differences (Additional file 9).

**Figure 6:**
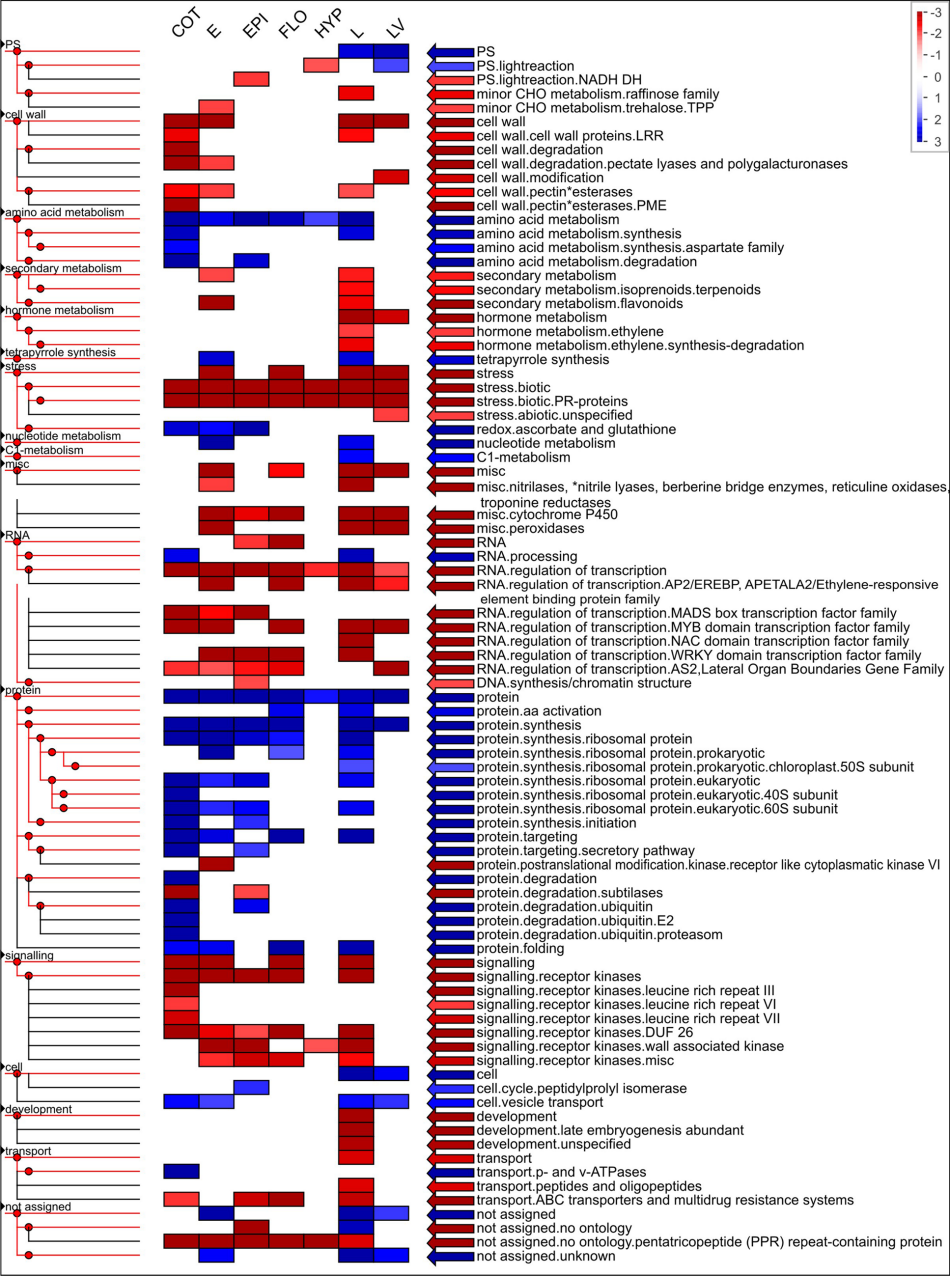
**Enriched terms of MapMan categories for all different tissues and treatments**; enrichment was tested as described for Figure 4.

After establishing that the difference in growth conditions of seedlings had a profound effect on the pathway pattern, all tissue samples were compared (Figure 6). Mature leaves were enriched in photosynthesis and the protein synthesis categories as well as the cell and unassigned categories. This pattern reflected the major commitment of a mature leaf to photosynthesis and the protein turnover that was associated with the oxidative load on the chloroplasts, which was compensated by protein synthesis. In contrast, in young cotyledons, which were, unlike in *Arabidopsis*, storage organs for seedling development, photosynthesis was not an enriched category. Since the pea cotyledons mobilized their reserves, especially proteins [62], the library was enriched in protein and especially its subcategories of protein degradation and in amino acid metabolism. In addition, the cotyledon library was enriched in cell/vesicle transport and p- and v-ATPases as may be expected for cells turning over protein stored in vesicles [62]. The hypocotyl library had only amino acid metabolism and protein biosynthesis overrepresented. Possibly, young tissues as those sampled for the libraries were all overrepresented with regard to protein and amino acid metabolism, since both are required to maintain cell division and multiplication typical of young tissues.

Taken together, the pathway representation analysis validated the strategy to sample multiple aerial tissues to achieve good coverage of pathways. Each of the single libraries contributed sequences corresponding to 8515 to 13298 AGIs presenting 50% to 78% of the 17104 AGIs identified by the union of all libraries (Table 4).

## Conclusions

The application of next generation sequencing to the transcriptome of *Pisum sativum*, the garden pea, resulted in 450 Megabases of transcriptome sequence derived from above-ground organs of pea. Comparison to Arabidopsis and mathematical analysis showed that the transcript coverage was near complete and revealed the effects of normalization on sequence yield and gene content. The pathway representation analysis also showed that the different libraries used for sequencing have different pathway signatures, which fit biological expectations. Based on the analysis of the transcriptome resources the pea can now be treated as a biochemical model plant with near complete transcript coverage regarding different aerial tissues. The usefulness of the database in a preliminary form has already been demonstrated for organelle proteomics [5,24]. Although the data was assembled with programs used by other transcriptome NGS projects [16-20], the assembly itself was revealed to be a major bottleneck. 454 sequencing technology followed by read quantification and read assembly were successfully used not only for the study of non-model legumes but also for the analysis of the C_4 _syndrome [46,63].

Completing the transcriptome coverage for pea will require not only sequencing of libraries derived from below ground organs and various stages of seed development but also improvement in assembly technology.

## Methods

### Plant material and treatment

Pea seeds of the variety 'Little Marvel' were purchased from a commercial supplier and seeds were sown in soil and grown under cool light fluorescent lamps for two weeks. All cDNA libraries reported in this study were made from plants grown from the same commercial batch of seeds. Green leaves and flowers were harvested and plunged immediately into liquid nitrogen. Yellow etiolated leaves were harvested as above from plants grown in the dark. De-etiolated leaves were harvested from the dark grown plants after exposure to the cool fluorescent lamps for 6 hours. The epicotyls and hypocotyls were harvested from 6 days-old pea seedlings germinated on a moist filter paper in dark. Cotyledons were harvested after removing epicotyls and hypocotyls.

### RNA extraction

Total RNA extraction and synthesis of double stranded cDNA was performed as described previously [64]. Briefly, one gram of pea plant samples were ground with a mortar and pestle in liquid nitrogen and total RNA was extracted in guanidinium thiocyanate-phenol-chloroform mixture and pelleted, followed by two washes of the RNA pellet with 3 M sodium acetate (pH 6.0). The quality of the isolated RNA was analyzed using formaldehyde agarose gel electrophoresis and the Agilent 2100 Bioanalyzer RNA chip (Agilent Technologies, CA). The mRNA was isolated using the PolyATract mRNA isolation system (Promega, WI) and concentrated by precipitation with ethanol.

### cDNA library preparation

The preparation of cDNA libraries was conducted as described previously [64]: The cDNA was synthesized using the Smart PCR cDNA synthesis kit according to the manufacturers suggestions (Clontech, CA) using 1 mg mRNA. Double-strand cDNA was prepared from 2 mL of the first-strand reaction by PCR (13 cycles). The cDNA was purified using QIAquick PCR purification spin columns (Qiagen) and was checked for purity and degradation using the Agilent 2100 Bioanalyzer DNA chip.

### Normalization of cDNA library

Some cDNA libraries were normalized to decrease the amount of highly abundant transcripts. To this end, 1 μg of double-stranded cDNA was normalized using a commercial kit (Trimmer-kit, Evrogen, Moscow, Russia) that is based on Kamchatka crab duplex-specific nuclease. The normalization efficiency was analyzed by quantitative PCR using primers for Rubisco small subunit (highly redundant) and CP12 (moderately redundant).

Following normalization, the double stranded cDNA was PCR amplified, quality checked with agarose gels and the Agilent's Bioanalyzer DNA chip and 3 μg of normalized cDNA was used for sequencing with a GS 20 or GS FLX sequencer (Roche/454 Life Sciences, CT), respectively.

### EST pre-processing and assembly

Sequence reads obtained from 454 software were cleaned from cDNA primer contaminations using crossmatch [28] and an in house python script clipped the masked contaminations, discarded reads shorter than 50 nts and adjusted the quality files accordingly. The cleaned reads were assembled with 1,198 partial and complete coding sequences from *Pisum sativum *downloaded from NCBI in a hybrid assembly using MIRA [25,29]. MIRA parameters used were: de novo, est, accurate, sanger, 454 including polyA/T clipping for 454 ESTs. Besides singletons that MIRA evaluated as high confidence singletons, MIRA discards many singletons during the assembly to a separate debris file. Of these debris singletons all with a significant BlastX hit against the *A. thaliana *proteome (TAIR9) [65] were added to subsequent analyses.

A second pass assembly using all the 324,428 unigenes obtained by the previous MIRA assembly was performed with the TGICL clustering and assembly pipeline including CAP3 [31,32]. Both programs were run with default parameter settings, requiring 94% sequence identity, a minimum of 40 nucleotides overlap and a maximal overhang of 30 nucleotides for assembly. As the largest three clusters that were produced by TGICL could not be assembled with CAP3 due to memory limitations, they were additionally preclustered and assembled with scripts provided within the TGICL pipeline with default parameter settings [32].

### Unigene annotation

The unigenes were annotated by queries against the proteomes of *A. thaliana *(TAIR9), *M. trunculata *(version 3.0), *G. max *(version1.0) [50,51,65] and the non redundant protein database from NCBI [13] using BlastX (e-value ≤ 10^-4^). Functional annotation of the unigenes was done with MapMan categories [54] and gene ontology terms [58] via the AGI annotation.

### Quantification of different sources contributing to redundancy between unigenes

Orthologous cDNA sequences from *P. sativum *to the most frequent AGIs used for the annotation of the first pass MIRA unigenes (Table 2) where retrieved from NCBI [13]. This yielded four complete and one partial coding sequence: (Lhcb1) light-harvesting chlorophyll-a/b binding protein, (Lhcb2) light-harvesting chlorophyll-a/b binding protein, (RuBP) ribulose 1,5 bisphosphate carboxylase, (Ran1) Ran1 and (Ccbp) chloroplast chlorophyll-a/b binding protein. Five genes published as single copy genes from pea [36-40] and four genes encoding Mendelian traits [41-44] were added to the analysis. All 324,428 unigenes from the first pass MIRA assembly were mapped against the retrieved reference sequences using the BWA-SW Aligner [66]. The column wise information of the alignments was read out employing SAM tools [67] and custom written python scripts. For each alignment the number of alignment positions (columns) with the following characteristics was read out: (i) identical nucleotides for all unigenes at that position, (ii) coverage by at least 25 identical variants (identical point mutations) within the unigenes, (iii) coverage by at least 4 insertions/deletions of a nucleotide length dividable by three and (iv) coverage by at least one variant (point mutation or insertions/deletions of a length not dividable by three) but not exceeding the required threshold of 25 identical variants.

### Mathematical analysis of library completeness

To test the transcript coverage of reads from a specific library/the combination of all libraries mathematical analysis similar to rarefaction analysis was employed. For that purpose a read pool was defined, i.e. all reads obtained from one library or all reads from all libraries combined. From such a read pool reads were randomly drawn to create different sets of reads with increasing sample sizes. For each of those given sets the number of *Arabidopsis *genes that could be identified by the reads within the set was recorded with the size of the read set. This process was automated in a python script. The identification of the AGI by a read was done via the MIRA unigenes and their best BlastX hit against the *Arabidopsis *proteome (TAIR9) [65]. The numbers of identified *Arabidopsis *genes were plotted against the corresponding sample sizes and the data points were fitted by non-linear regression with the model y = ax/(b+x) (SigmaPlot software, Systat Software Inc - Scientific Software Products). If the sequencing of transcripts from a particular tissue was exhaustive, the resulting curve was expected to "saturate"; it converged against a fixed value, parameter "a" in the model function indicating an upper limit for gene detection. This "saturation" was also represented by a decreasing slope at higher sample sizes, which indicated decreasing potential to detect additional *Arabidopsis *genes when further sampling from the defined read pool. An identical approach was also taken to approximate the transcript coverage with the difference that the number of all identified AGIs was not recorded but the number of AGIs that was identified at least by 5, 10 and 100 reads.

### Enrichment analysis

All enrichment tests were performed based on *A. thaliana *annotation of the unigenes. Enrichment analysis with MapMan categories [54] was tested with Fisher's exact test, using the PageMan application [53] and multiple testing was corrected for by FDR (Benjamini-Hochberg). The gene test sets always consisted of all Arabidopsis genes identified by the unigenes present in that library/tissue type. As the reference gene set for the analysis all *Arabidopsis *genes identified with reads of any library important for a specific question were chosen. These were the following library combinations: union of all genes in all normalized and non-normalized leaf libraries (Figure 4), union of all genes in libraries E and L (Figure 5) and union of all genes from all obtained libraries (Figure 6).

Enrichment of GO terms was tested with Fisher's exact test, using the Bioconductor package topGO version 0.9.7 [58,59]. The weight01 algorithm of topGO was used accounting for local dependencies within the graph structure of the gene ontology.

### Accession numbers of raw data

The sequence read data reported in this manuscript have been deposited in the NCBI Sequence Read Archive and are available under the Accession Number [NCBI:SRA031288]. The initial MIRA assembly reported in this manuscript has been modified according to NCBI guidelines and deposited in the NCBI Transcriptome Shotgun Assembly Archive and is available under the Accession Number [JI896856 - JI981123].

## Competing interests

The authors declare that they have no competing interests.

## Authors' contributions

SUF performed the bioinformatics analysis, analyzed the data and co-wrote the manuscript, RPS generated the sequencing libraries, AB analyzed the data and wrote the manuscript, EBB participated in the bioformatics analysis and helped draft the manuscript and APMW conceived of the study, and participated in its design and coordination and helped to draft the manuscript. All authors read and approved the final manuscript.

## Supplementary Material

Additional file 1 **Fasta file of the unigene database for the the second pass TGICL/CAP3 assembly using the MIRA unigenes of the first pass assembly**; The fasta file contains the Mira unigenes annotated with their best BlastX hit (e-value ≤ 10^-4^) against the proteome of *A. thaliana*, *M. truncatula*, *Glycine max *and the nr database of NCBI (with only the best hit against one proteome in the given order is presented). Click here for file

Additional file 2 **Mapping of the cleaned reads, the first pass assembly and the second pass assembly results against *A. thaliana*, *M. truncatula *and *G. max *to approximate the proportion of chimeric genes in the assembly**. Click here for file

Additional file 3 **Contribution of retrotransposon-like sequences to the contig databases**. Click here for file

Additional file 4 **The alignments used to generate **Table 3**; data can be loaded into tablet **[52]**for visualization**. Click here for file

Additional file 5 **The fasta file of sequences used to generate **Table 3**; data can be loaded into tablet **[45]**for visualization**. Click here for file

Additional file 6 **Correlation between reads mapping to a reference and unigenes mapping to the same reference**. Click here for file

Additional file 7 **Rarefaction analysis of gene representation in different libraries; analysis was performed as described in Figure **3**(A) Reads were randomly sampled from the COT library, (B) Reads were randomly sampled from the FLO library, (C) Reads were randomly sampled from the HYP library, (D) Reads were randomly sampled from the EPI library, (E) Reads were randomly sampled from the either of the different normalized leaf libraries (LVN.1-5), (F) Reads were randomly sampled from the library made from etiolated seedlings (E) or the library made from etiolated seedlings after light treatment (L)**. Click here for file

Additional file 8 **Proteome coverage of 3' vs. 5' ends; Different sets of query sequences were blasted against the complete *Arabidopsis *proteome, TAIR9 pep (black) and only 5', 3' ends (100 end standing amino acids) of all peptides, (red, green, respectively) and the number of significant hits (BlastX, e-value ≤ 10^-4^) was recorded**. The sets of query sequences were all first pass MIRA unigenes (unigenes) and all cleaned reads from the different libraries (COT, E, L, EPI, LVN.5, LVN.1, LVN.2, LVN.3, LVN.4, FLO, HYP, LVR.1). Click here for file

Additional file 9 **Enriched categories of GO terms for libraries of etiolated seedlings and etiolated seedlings after light treatment; enrichment analysis was performed with topGO **[64]. Click here for file
