# Supplementary figures and images for: Comprehensive transcriptome analysis of the highly complex Pisum sativum genome using next generation sequencing
Source: BMC Genomics. 2011 May 11;12:227. doi: 10.1186/1471-2164-12-227 (PMC3224338; doi:10.1186/1471-2164-12-227)

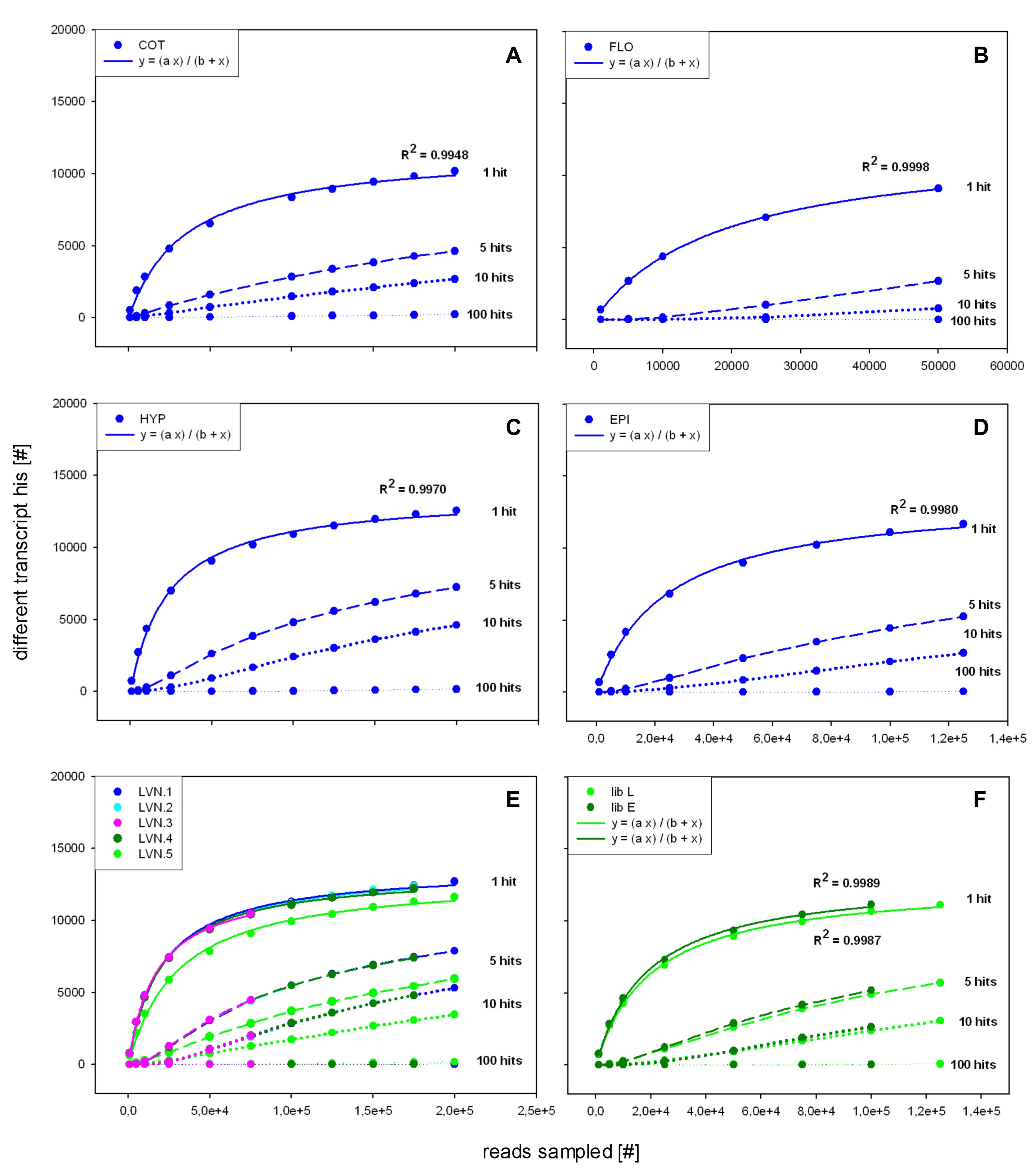

Supplement: Additional file 7 — Rarefaction analysis of gene representation in different libraries; analysis was performed as described in Figure 3(A) Reads were randomly sampled from the COT library, (B) Reads were randomly sampled from the FLO library, (C) Reads were randomly sampled from the HYP library, (D) Reads were randomly sampled from the EPI library, (E) Reads were randomly sampled from the either of the different normalized leaf libraries (LVN.1-5), (F) Reads were randomly sampled from the library made from etiolated seedlings (E) or the library made from etiolated seedlings after light treatment (L). [file 1471-2164-12-227-S7.JPEG]

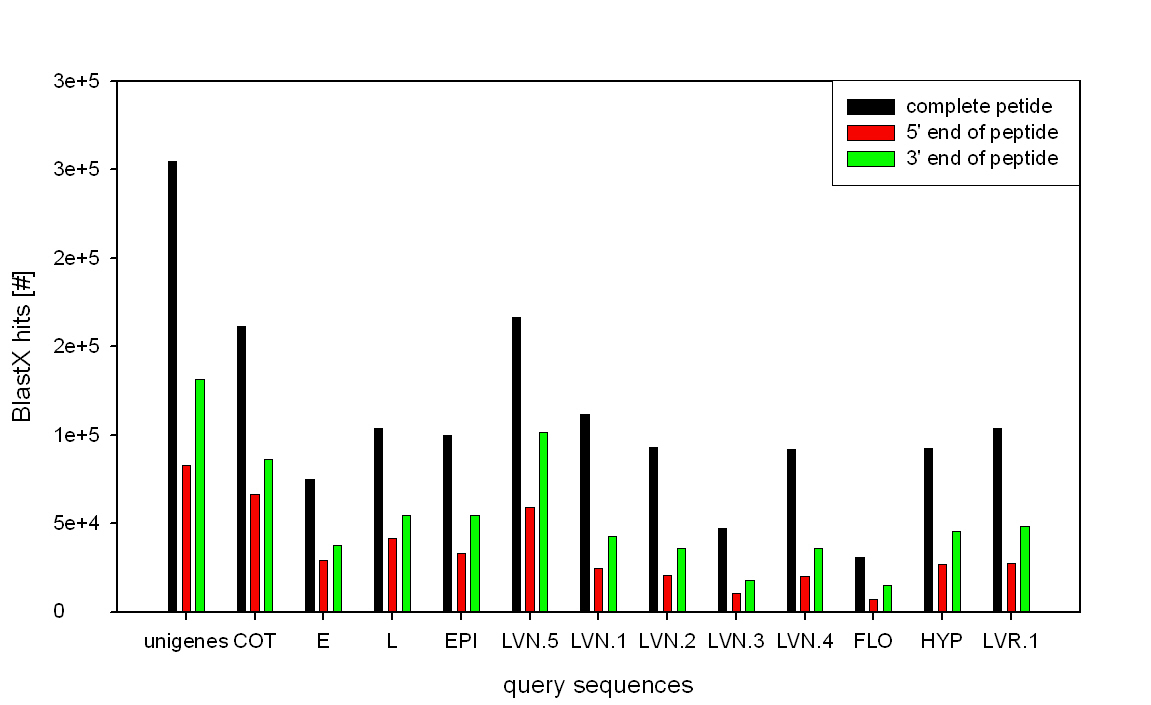

Supplement: Additional file 8 — Proteome coverage of 3' vs. 5' ends; Different sets of query sequences were blasted against the complete Arabidopsis proteome, TAIR9 pep (black) and only 5', 3' ends (100 end standing amino acids) of all peptides, (red, green, respectively) and the number of significant hits (BlastX, e-value ≤ 10-4) was recorded. The sets of query sequences were all first pass MIRA unigenes (unigenes) and all cleaned reads from the different libraries (COT, E, L, EPI, LVN.5, LVN.1, LVN.2, LVN.3, LVN.4, FLO, HYP, LVR.1). [file 1471-2164-12-227-S8.JPEG]
