# Supplementary material for: Comprehensive transcriptome analysis of the highly complex Pisum sativum genome using next generation sequencing
Source: BMC Genomics. 2011 May 11;12:227. doi: 10.1186/1471-2164-12-227 (PMC3224338; doi:10.1186/1471-2164-12-227)
Supplement: Additional file 9 — Enriched categories of GO terms for libraries of etiolated seedlings and etiolated seedlings after light treatment; enrichment analysis was performed with topGO [64]. [file 1471-2164-12-227-S9.PDF]

1 Enrichment analysis with topGO (using GO-terms as backend database)

1.1 Enrichment analysis with libraries E and L

Enrichment of GO terms for the three different ontologies (cellular component, Biological process, molecular function) was tested with topGO for libraries of etiolated seedlings (E) and etiolated seedlings after light treatment (L). For the different libraries and ontologies always the 20 GO terms with the lowest p-values according to the weight01 algorithm of topGO are shown if available. Additionally the classical p-values according to Fisher's exact test without multiple testing correction are provided.

Table 1: Results of enrichment analysis using topGO with: selected genes (sel) = all AGIs present in library L, background genes (bg) = all AGIs present in the union of libraries E and L, ontology used = cellular component

	GO.ID	term	counts bg	counts sel	expected	rank in classic	p- val classic	p-val weight
1	GO:0009570	chloroplast stroma	357	349	316.08	23	4.8e-11	7.0e-11
2	GO:0009941	chloroplast envelope	384	375	339.99	22	1.6e-11	9.7e-10
3	GO:0009535	chloroplast thylakoid membrane	274	268	242.59	25	7.7e-09	2.3e-08
4	GO:0009507	chloroplast	2307	2154	2042.57	14	5.0e-18	1.0e-05
5	GO:0005783	endoplasmic reticulum	277	265	245.25	42	2.1e-05	0.00012
6	GO:0005730	nucleolus	218	208	193.01	46	0.00030	0.00030
7	GO:0005739	mitochondrion	722	675	639.25	37	2.7e-06	0.00059
8	GO:0005737	cytoplasm	4439	4117	3930.21	1	$< 1e-30$	0.00097
9	GO:0048046	apoplast	183	174	162.02	56	0.00167	0.00167
10	GO:0005886	plasma membrane	1476	1339	1306.82	57	0.00230	0.00438
11	GO:0009579	thylakoid	386	375	341.76	24	3.2e-10	0.00867
12	GO:0010287	plastoglobule	56	55	49.58	68	0.00892	0.00892
13	GO:0005777	peroxisome	129	123	114.21	61	0.00596	0.00983
14	GO:0022627	cytosolic small ribosomal subunit	62	60	54.89	75	0.02123	0.02123
15	GO:0010319	stromule	31	31	27.45	76	0.02284	0.02284
16	GO:0009706	chloroplast inner membrane	36	35	31.87	92	0.07042	0.02287
17	GO:0022625	cytosolic large ribosomal subunit	87	83	77.03	78	0.02301	0.02301
18	GO:0016020	membrane	2967	2708	2626.93	30	1.6e-08	0.02444
19	GO:0009536	plastid	2357	2203	2086.84	13	3.4e-19	0.03329
20	GO:0005773	vacuole	420	383	371.86	84	0.04480	0.04968

Table 2: Results of enrichment analysis using topGO with: selected genes (sel) = all AGIs present in library L, background genes (bg) = all AGIs present in the union of libraries E and L, ontology used = biological process

	GO.ID	term	counts bg	counts sel	expected	rank in classic	p- val classic	p-val weight
1	GO:0006457	protein folding	180	173	158.85	8	0.00017	8.6e-05
2	GO:0006412	translation	407	386	359.17	2	2.9e-06	0.00021
3	GO:0046686	response to cadmium ion	234	222	206.5	12	0.00038	0.00038
4	GO:0006396	RNA processing	223	216	196.79	3	3.2e-06	0.00888
5	GO:0009853	photorespiration	28	28	24.71	81	0.03005	0.03005
6	GO:0015986	ATP synthesis coupled proton transport	27	27	23.83	84	0.03406	0.03406
7	GO:0009651	response to salt stress	255	235	225.03	77	0.02664	0.03691
8	GO:0015995	chlorophyll biosynthetic process	32	30	28.24	438	0.25690	0.04972
9	GO:0006364	rRNA processing	30	30	26.47	73	0.02338	0.06360
10	GO:0010090	trichome morphogenesis	34	32	30	376	0.22017	0.06380
11	GO:0042254	ribosome biogenesis	112	107	98.84	38	0.00664	0.07809
12	GO:0006096	glycolysis	38	37	33.53	109	0.05212	0.07839
13	GO:0009407	toxin catabolic process	20	20	17.65	160	0.08186	0.08186
14	GO:0009744	response to sucrose stimulus	33	32	29.12	169	0.08682	0.09595
15	GO:0006470	protein amino acid dephosphorylation	46	44	40.59	154	0.08060	0.09600
16	GO:0006950	response to stress	1108	984	977.78	486	0.28992	0.10504
17	GO:0006414	translational elongation	21	21	18.53	145	0.07223	0.10510
18	GO:0009793	embryonic development ending in seed dor...	273	249	240.92	143	0.07058	0.11589
19	GO:0016192	vesicle-mediated transport	165	153	145.61	92	0.04040	0.11741
20	GO:0006397	mRNA processing	55	53	48.54	88	0.03546	0.11871

Table 3: Results of enrichment analysis using topGO with: selected genes (sel) = all AGIs present in library L, background genes (bg) = all AGIs present in the union of libraries E and L, ontology used = molecular function

	GO.ID	term	counts bg	counts sel	expected	rank in classic	p- val classic	p-val weight
1	GO:0003735	structural constituent of ribosome	255	241	224.37	4	0.00031	0.00031
2	GO:0003723	RNA binding	365	342	321.16	2	0.00017	0.00438
3	GO:0003824	catalytic activity	4686	4160	4123.19	17	0.01800	0.00656
4	GO:0051082	unfolded protein binding	76	73	66.87	9	0.01433	0.01433
5	GO:0003755	peptidyl-prolyl cis-trans isomerase acti...	49	48	43.11	10	0.01442	0.01442
6	GO:0030528	transcription regulator activity	786	651	691.6	1839	1.00000	0.02657
7	GO:0003924	GTPase activity	55	53	48.39	21	0.03151	0.03151
8	GO:0004091	carboxylesterase activity	122	115	107.35	14	0.01606	0.03771
9	GO:0051287	NAD or NADH binding	25	25	22	22	0.04067	0.04067
10	GO:0031072	heat shock protein binding	87	82	76.55	24	0.04157	0.04157
11	GO:0008026	ATP-dependent helicase activity	85	80	74.79	30	0.04836	0.04779
12	GO:0005525	GTP binding	138	128	121.43	28	0.04806	0.04806
13	GO:0008233	peptidase activity	110	100	96.79	116	0.21602	0.05296
14	GO:0003746	translation elongation factor activity	22	22	19.36	34	0.05975	0.05975
15	GO:0004364	glutathione transferase activity	22	22	19.36	35	0.05975	0.05975
16	GO:0003676	nucleic acid binding	1717	1507	1510.78	439	0.63677	0.06516
17	GO:0005488	binding	5173	4555	4551.7	236	0.43734	0.07543
18	GO:0005528	FK506 binding	20	20	17.6	44	0.07721	0.07721
19	GO:0051536	iron-sulfur cluster binding	34	33	29.92	41	0.07250	0.11322
20	GO:0005200	structural constituent of cytoskeleton	17	17	14.96	64	0.11341	0.11341

Table 4: Results of enrichment analysis using topGO with: selected genes (sel) = all AGIs present in library E, background genes (bg) = all AGIs present in the union of libraries E and L, ontology used = cellular component

	GO.ID	term	counts bg	counts sel	expected	rank in classic	p- val classic	p-val weight
1	GO:0009570	chloroplast stroma	357	347	314.68	9	3.5e-10	1.2e-10
2	GO:0009535	chloroplast thylakoid membrane	274	264	241.52	24	1.2e-06	1.1e-06
3	GO:0005737	cytoplasm	4439	4051	3912.84	1	5.0e-17	2.6e-05
4	GO:0009941	chloroplast envelope	384	363	338.48	32	1.3e-05	2.6e-05
5	GO:0005794	Golgi apparatus	184	175	162.19	37	0.00094	0.0035
6	GO:0005886	plasma membrane	1476	1329	1301.05	50	0.00800	0.0041
7	GO:0005829	cytosol	515	481	453.96	33	3.7e-05	0.0042
8	GO:0005773	vacuole	420	385	370.22	52	0.01137	0.0045
9	GO:0048046	apoplast	183	172	161.31	49	0.00590	0.0059
10	GO:0009543	chloroplast thylakoid lumen	65	63	57.3	53	0.01282	0.0128
11	GO:0009505	plant-type cell wall	146	137	128.69	57	0.01623	0.0162
12	GO:0022625	cytosolic large ribosomal subunit	87	83	76.69	58	0.01791	0.0179
13	GO:0005730	nucleolus	218	202	192.16	59	0.01930	0.0193
14	GO:0010319	stromule	31	31	27.33	60	0.01991	0.0199
15	GO:0005739	mitochondrion	722	660	636.42	45	0.00222	0.0221
16	GO:0009579	thylakoid	386	369	340.25	19	2.1e-07	0.0306
17	GO:0005618	cell wall	340	316	299.7	44	0.00219	0.0404
18	GO:0045271	respiratory chain complex I	38	37	33.5	70	0.05031	0.0503
19	GO:0009507	chloroplast	2307	2104	2033.55	15	1.0e-07	0.0624
20	GO:0005840	ribosome	302	284	266.2	35	0.00038	0.0712

Table 5: Results of enrichment analysis using topGO with: selected genes (sel) = all AGIs present in library E, background genes (bg) = all AGIs present in the union of libraries E and L, ontology used = biological process

	GO.ID	term	counts bg	counts sel	expected	rank in classic	p- val classic	p-val weight
1	GO:0046686	response to cadmium ion	234	226	205.8	1	2.5e-06	2.5e-06
2	GO:0006412	translation	407	380	357.96	7	0.00018	0.00032
3	GO:0009651	response to salt stress	255	240	224.27	10	0.00068	0.00091
4	GO:0006979	response to oxidative stress	149	139	131.04	61	0.02325	0.00161
5	GO:0006511	ubiquitin-dependent protein catabolic pr...	164	156	144.24	13	0.00140	0.00192
6	GO:0042254	ribosome biogenesis	112	106	98.5	49	0.01398	0.00241
7	GO:0009409	response to cold	174	164	153.03	21	0.00403	0.00684
8	GO:0009965	leaf morphogenesis	79	76	69.48	42	0.01055	0.01619
9	GO:0016051	carbohydrate biosynthetic process	173	156	152.15	307	0.21871	0.03103
10	GO:0009737	response to abscisic acid stimulus	166	154	146	69	0.02973	0.04129
11	GO:0009753	response to jasmonic acid stimulus	76	74	66.84	20	0.00367	0.04934
12	GO:0016481	negative regulation of transcription	39	37	34.3	190	0.13498	0.05915
13	GO:0006888	ER to Golgi vesicle-mediated transport	22	22	19.35	104	0.05915	0.05915
14	GO:0009555	pollen development	72	65	63.32	469	0.34891	0.06058
15	GO:0006970	response to osmotic stress	276	261	242.74	5	0.00014	0.08443
16	GO:0009867	jasmonic acid mediated signaling pathway	26	26	22.87	75	0.03535	0.08688
17	GO:0009733	response to auxin stimulus	135	124	118.73	147	0.09800	0.09992
18	GO:0007568	aging	54	53	47.49	35	0.00808	0.10750
19	GO:0010150	leaf senescence	17	17	14.95	166	0.11254	0.11254

Table 6: Results of enrichment analysis using topGO with: selected genes (sel) = all AGIs present in library E, background genes (bg) = all AGIs present in the union of libraries E and L, ontology used = molecular function

	GO.ID	term	counts bg	counts sel	expected	rank in classic	p- val classic	p-val weight
1	GO:0003735	structural constituent of ribosome	255	241	224.31	2	0.00030	0.00030
2	GO:0003723	RNA binding	365	335	321.07	5	0.01125	0.00817
3	GO:0005198	structural molecule activity	331	313	291.16	1	3.2e-05	0.02318
4	GO:0015450	P-P-bond-hydrolysis-driven protein trans...	33	33	29.03	7	0.01444	0.02412
5	GO:0042803	protein homodimerization activity	39	38	34.31	12	0.04241	0.04241
6	GO:0004842	ubiquitin-protein ligase activity	156	144	137.22	17	0.05393	0.05393
7	GO:0003755	peptidyl-prolyl cis-trans isomerase acti...	49	47	43.1	18	0.05513	0.05513
8	GO:0015171	amino acid transmembrane transporter act...	33	31	29.03	79	0.22331	0.06759
9	GO:0005525	GTP binding	138	127	121.39	24	0.08422	0.08422
10	GO:0005507	copper ion binding	68	64	59.82	23	0.07563	0.09474
11	GO:0004712	protein serine/threonine/tyrosine kinase...	37	35	32.55	53	0.16074	0.10868
12	GO:0008266	poly(U) RNA binding	16	16	14.07	38	0.12833	0.12833
13	GO:0010181	FMN binding	16	16	14.07	39	0.12833	0.12833
14	GO:0046961	proton-transporting ATPase activity & rot...	16	16	14.07	40	0.12833	0.12833
15	GO:0016762	xyloglucan:xyloglucosyl transferase acti...	15	15	13.19	49	0.14591	0.14591
16	GO:0016168	chlorophyll binding	26	25	22.87	54	0.16215	0.16215
17	GO:0008094	DNA-dependent ATPase activity	24	23	21.11	68	0.19704	0.16586
18	GO:0004028	3-chloroallyl aldehyde dehydrogenase act...	14	14	12.32	55	0.16590	0.16590
19	GO:0030145	manganese ion binding	14	14	12.32	56	0.16590	0.16590
20	GO:0000166	nucleotide binding	1342	1174	1180.48	607	0.73474	0.17521
